# Interacting and joint effects of atherogenic index of plasma and dietary inflammatory index on hypertension-diabetes comorbidity risk in Chinese elderly adults: results from China Nutrition and Health Surveillance in 2015–2017

**DOI:** 10.3389/fnut.2026.1786023

**Published:** 2026-03-27

**Authors:** Xingxing Wu, Hongyun Fang, Bulai Lu, Huijun Wang, Wenwen Du, Shujuan Li, Qiya Guo, Xiaoqi Wei, Weiyi Gong, Lei Hua, Lahong Ju

**Affiliations:** 1 National Institute for Nutrition and Health, Chinese Center for Disease Control and Prevention, Beijing, China; 2 Key Laboratory of Public Nutrition and Health, National Health Commission of the People's Republic of China, Beijing, China; 3 National Key Laboratory of Intelligent Tracking and Forecasting for Infectious Diseases, Public Health Emergency Center, Chinese Center for Disease Control and Prevention, Beijing, China

**Keywords:** atherogenic index of plasma, China, dietary inflammatory index, hypertension-diabetes comorbidity, older adults

## Abstract

**Objective:**

This study aimed to investigate the interaction and joint effects of the atherogenic index of plasma (AIP) and dietary inflammatory index (DII) on hypertension-diabetes comorbidity (HDC) among Chinese elderly adults.

**Methods:**

The study data were derived from the 2015–2017 China Nutrition and Health Surveillance. Participants were categorized by AIP tertiles (T1, T2, T3) and DII score (<0, ≥0). Logistic regression models were used to analyze the independent associations, interactions, and joint effects of AIP and DII on HDC.

**Results:**

A total of 18,447 elderly participants were included, with a median age of 66.32 (IQR: 62.86, 71.56) years and including 9,650 (52.31%) males. The median AIP was −0.04 (IQR: −0.24, 0.17), and the median DII score was 1.63 (IQR: −0.13, 2.85). After adjusting for confounders, compared with the T1 group of AIP, the risk of HDC increased by 0.37-fold (OR = 1.37, 95% CI: 1.14–1.65, *p* = 0.001) in the T2 group and by 1.30-fold (OR = 2.30, 95% CI: 1.93–2.74, *p* < 0.001) in the T3 group; compared with the anti-inflammatory diet group (DII < 0), the risk of HDC increased by 0.19-fold (OR = 1.19, 95% CI: 1.03–1.38, *p* = 0.019) in the pro-inflammatory diet group (DII ≥ 0). No significant multiplicative (OR = 0.94, 95% CI: 0.63–1.40) and additive (relative excess risk due to interaction = 0.25, 95% CI: −0.37 − 0.87) interactions were found between AIP and DII on the risk of HDC. Results of the joint effect analysis showed that, compared with the group with an anti-inflammatory diet and lower AIP, the group with a pro-inflammatory diet and higher AIP had a 1.98-fold (OR = 2.98, 95% CI: 2.16–4.13, *p* < 0.001) increased risk of HDC.

**Conclusion:**

Higher AIP levels and a pro-inflammatory diet were both associated with an increased risk of hypertension-diabetes comorbidity in Chinese elderly adults. Although the multiplicative and additive interactions between AIP and DII on HDC risk were not statistically significant, joint effect analysis revealed that individuals with both high AIP and a pro-inflammatory diet had a significantly higher HDC risk compared to those with only a single risk factor.

## 1 Introduction

Hypertension and diabetes share significant overlaps in etiology and disease mechanisms (1–3), and they frequently coexist. Existing studies indicate that the simultaneous presence of hypertension and diabetes (i.e., hypertension-diabetes comorbidity, HDC) poses a higher health risk compared to having neither nor only one of these diseases (4, 5). In Japanese and Western populations, approximately 50% of diabetic patients have hypertension, and 20% of hypertensive patients have diabetes (6). Data from the 2008–2011 Korea National Health and Nutrition Examination Survey showed that the prevalence of HDC among individuals aged 50 and above was 11.27% in men and 10.05% in women (7). The 2013 US National Inpatient Sample database reported an HDC prevalence of 28.53% among the elderly aged 65 and above (8). Data from the 2018 China Chronic Disease and Risk Factor Surveillance indicated an HDC prevalence of 17.7% among the elderly aged 65–75 (9), suggesting a difficult situation of HDC among the elderly.

The atherogenic index of plasma (AIP) was proposed by Dobiásová and Frohlich (10) in 2001 as a novel marker for assessing plasma atherogenicity. Recent studies have shown that elevated AIP is positively associated with HDC risk (11, 12). However, research on the association between AIP and HDC specifically in the Chinese elderly population remains limited. Meanwhile, diet is one of the modifiable lifestyle factors that can regulate the body’s inflammatory status (13). The dietary inflammatory index (DII) is commonly used to assess the inflammatory potential of an individual’s diet; a lower DII score indicates a stronger anti-inflammatory effect, while a higher score indicates a stronger pro-inflammatory effect (14). Studies have shown that a higher DII may be a risk factor for hypertension (15, 16) and diabetes (17). Furthermore, research has found a positive correlation between DII and AIP (18), suggesting a potential synergistic mechanism between DII and AIP.

Currently, research on the independent and joint effects of AIP and DII on HDC remains limited, especially in the Chinese elderly population. Therefore, this study aims to investigate the interaction and joint effects of AIP and DII on HDC in the Chinese elderly population, to provide a scientific basis for the prevention and control of HDC.

## 2 Methods

### 2.1 Data source and survey participants

This study used data from the 2015–2017 China Nutrition and Health Surveillance (CNHS) project. A multi-stage cluster random sampling method was employed to select a representative sample from 31 provinces (autonomous regions/municipalities) in mainland China. For more details, please refer to reference (19).

Inclusion criteria for this analysis were: (1) age ≥ 60 years; (2) complete data on demographic information, dietary survey, medical examination, and laboratory tests.

Exclusion criteria were: (1) unreasonable dietary energy intake (< 800 kcal/d or > 5,000 kcal/d); (2) a previous diagnosis of hypertension, diabetes, or dyslipidemia that led to dietary intervention. This was determined based on participants’ responses to the survey question: “Have you ever been diagnosed with hypertension, diabetes, or dyslipidemia by a physician? If yes, have you adopted dietary control measures to manage your blood pressure, blood glucose, and/or blood lipids?” Participants who answered “yes” to adopting dietary control measures were excluded to minimize the impact of illness-related dietary changes on the results.

A total of 18,447 participants were finally included in this study. All participants provided written informed consent before the survey, and this study was approved by the Ethics Committee of the Chinese Center for Disease Control and Prevention (approval number: 201519-B).

### 2.2 Survey content

The 2015–2017 China Nutrition and Health Surveillance consisted of four parts: questionnaire interview, medical examination, laboratory testing, and dietary survey. Uniformly trained and qualified investigators collected household and individual basic information through face-to-face questionnaire interviews, including age, sex, place of residence, household economic income, education, marital status, smoking, alcohol consumption, physical activity, and family history of disease. Medical examinations included measurements of height, weight, waist circumference and blood pressure, conducted by uniformly trained investigators using measurement instruments certified and approved by the national project team, following standardized methods. Fasting venous blood (8 mL) was collected from all participants and sent to laboratories for centralized measurement of fasting blood glucose (FBG), hemoglobin A1c (HbA1c), triglycerides (TG), total cholesterol (TC), high-density lipoprotein cholesterol (HDL-C), and low-density lipoprotein cholesterol (LDL-C) according to national standards. Dietary surveys used a validated food frequency questionnaire (FFQ) (20) to collect information on the consumption frequency and amount of staple foods, legumes, vegetables, fruits, meat, aquatic products, eggs, dairy products, beverages, and alcohol over the past 12 months. The consumption of cooking oil and condiments in the household over the past month and the usual number of people eating at home per meal (breakfast, lunch, and dinner) were inquired to calculate per capita daily intake of cooking oil and condiments. Based on the Chinese Food Composition Table Standard Edition (Sixth Edition) (21), the daily average intake of energy and nutrients for participants was calculated.

### 2.3 Assessment of DII and AIP

Following the method proposed by Shivappa et al. (14), the DII score was used to measure the inflammatory potential of an individual’s diet. Although the original DII is based on 45 dietary components, based on the availability of dietary data in the CNHS database and the coverage of the Chinese Food Composition Table, 22 nutrients were finally included in this study to calculate the DII score for the elderly: energy, protein, carbohydrate, fat, saturated fatty acids, monounsaturated fatty acids, polyunsaturated fatty acids, dietary fiber, cholesterol, beta-carotene, vitamin A, vitamin B_1_, vitamin B_2_, niacin, folic acid, vitamin C, vitamin E, iron, zinc, selenium, magnesium, and alcohol. Referring to previous studies (22–24), participants were divided into DII < 0 (anti-inflammatory diet) and DII ≥ 0 (pro-inflammatory diet) groups based on the total DII score.

AIP was calculated using the formula AIP = log [TG (mmol/L)/HDL-C (mmol/L)] (10), with results rounded to two decimal places. Participants were divided into three groups based on AIP tertiles: T1 group (−0.92 ≤ AIP < −0.17), T2 group (−0.17 ≤ AIP < 0.09), and T3 group (0.09 ≤ AIP ≤ 1.33).

### 2.4 Outcome definition

HDC refers to participants simultaneously having hypertension and diabetes. The diagnostic criteria for hypertension were systolic blood pressure ≥ 140 mmHg and/or diastolic blood pressure ≥ 90 mmHg and/or self-report of a previous diagnosis of hypertension (25). The diagnostic criteria for diabetes were FBG ≥ 7.0 mmol/L and/or HbA1c ≥ 6.5% and/or self-report of a previous diagnosis of diabetes (26).

### 2.5 Covariates

Based on age, participants were categorized into five groups: 60–64, 65–69, 70–74, 75–79, and ≥80 years. Body mass index (BMI) was categorized according to Chinese standards (27): underweight (BMI < 18.5 kg/m^2^), normal (18.5 kg/m^2^ ≤ BMI < 24.0 kg/m^2^), overweight (24.0 kg/m^2^ ≤ BMI < 28.0 kg/m^2^), and obese (BMI ≥ 28.0 kg/m^2^). Place of residence was categorized as urban or rural. Education was categorized into three groups: primary school or below, middle school, and high school or above. Marital status was divided into “living with spouse” and “other status.” Annual household income per capita was categorized as < 4,000 CNY/year, 4,000–9,999 CNY/year, 10,000–19,999 CNY/year, ≥20,000 CNY/year, and missing. Smoking was categorized as never, former, and current smoker. Alcohol drinking was categorized as yes or no. Physical activity was classified into low (MET < 600), moderate (600 ≤ MET ≤ 3,000), and high (MET > 3,000) based on the weekly total metabolic equivalent (MET) of different activity levels and weekly total duration (28). Family history of hypertension or diabetes was defined as at least one parent, grandparent, or sibling diagnosed with the corresponding condition.

### 2.6 Statistical analyses

Data were described as means and standard deviations for normally distributed continuous variables, and as medians and interquartile ranges (IQR) for non-normally distributed continuous variables. Categorical variables were described as numbers and percentages, and group comparisons were conducted using the chi-square test. Multivariate logistic regression models were used to analyze the odds ratios (OR) and 95% confidence intervals (95% CI) for the association of AIP and DII with HDC. Model 1 was the crude model without adjustment for covariates. Model 2 was adjusted for age, sex, BMI, place of residence, education, marital status, and income. Model 3 was further adjusted for smoking, alcohol drinking, physical activity, family history of hypertension, and family history of diabetes based on Model 2. Additionally, restricted cubic spline (RCS) analysis was used to examine the relationship between AIP, DII, and HDC.

To quantify the interaction effects of AIP and DII on HDC, we followed established methodologies for interaction analysis (29–31). Following the approach described by VanderWeele and Knol (29), to analyze the interaction between two categorical exposure variables, interaction terms were constructed by focusing on two specific levels of each variable, specifically the product term of DII (anti-inflammatory and pro-inflammatory) and AIP (T1, T3). On a multiplicative scale, if the 95% CI of the OR for this product term did not include 1, it indicated a statistically significant multiplicative interaction. On an additive scale, interaction was assessed by calculating the relative excess risk due to interaction (RERI), the attributable proportion due to interaction (AP), and the synergy index (SI). The 95% CI for these three indicators was calculated using the delta method; when the 95% CI for RERI and AP did not include 0, and the 95% CI for SI did not include 1, the additive interaction was considered statistically significant.

To evaluate the joint effect of AIP and DII on HDC, participants were divided into 6 groups based on DII (anti-inflammatory/pro-inflammatory diet) and AIP (tertiles). Using multivariate logistic regression models, with the DII < 0 and lower AIP (T1) group as the reference, OR and their 95% CI were calculated for other groups.

To assess the robustness of our findings, we performed both sensitivity analyses using DII tertiles (T1: DII < 0.54; T2: 0.54 ≤ DII < 2.48; T3: DII ≥ 2.48) and stratified analyses by sex (male/female), repeating the main analysis steps for each.

All statistical analysis was performed using SAS 9.4 and R 4.5.0 software, with two-sided *p* values < 0.05 considered statistically significant.

## 3 Results

### 3.1 General characteristics of participants

This study ultimately included 18,447 participants, with a median age of 66.32 (IQR: 62.86, 71.56) years and including 9,650 (52.31%) males. The median AIP was −0.04 (IQR: −0.24, 0.17), and the median DII score was 1.63 (−0.13, 2.85). The general characteristics of participants by DII and AIP groups are shown in Table 1. The prevalence of HDC was 2.65% in the DII < 0 and lower AIP group and 9.87% in the DII ≥ 0 and higher AIP group, with a statistically significant difference (*p* < 0.001).

**Table 1:** General characteristics of elderly participants in CNHS 2015–2017.

Characteristics	DII < 0 and AIP < −0.17 (*n* = 1,586)	DII < 0 and AIP < 0.09 (*n* = 1,615)	DII < 0 and AIP ≥ 0.09 (*n* = 1,695)	DII ≥ 0 and AIP < −0.17 (*n* = 4,562)	DII ≥ 0 and AIP < 0.09 (*n* = 4,539)	DII ≥ 0 and AIP ≥ 0.09 (*n* = 4,450)	*P*-value
Age							<0.001
60–64	710 (44.77)	711 (44.02)	808 (47.67)	1701 (37.29)	1722 (37.94)	1926 (43.28)	
65–69	455 (28.69)	492 (30.46)	485 (28.61)	1,254 (27.49)	1,273 (28.05)	1,185 (26.63)	
70–74	247 (15.57)	239 (14.80)	245 (14.45)	794 (17.40)	766 (16.88)	705 (15.84)	
75–79	119 (7.50)	106 (6.56)	105 (6.19)	484 (10.61)	468 (10.31)	383 (8.61)	
≥80	55 (3.47)	67 (4.15)	52 (3.07)	329 (7.21)	310 (6.83)	251 (5.64)	
Sex							<0.001
Male	1,028 (64.82)	898 (55.60)	953 (56.22)	2,584 (56.64)	2,190 (48.25)	1997 (44.88)	
Female	558 (35.18)	717 (44.40)	742 (43.78)	1978 (43.36)	2,349 (51.75)	2,453 (55.12)	
BMI							<0.001
Underweight	113 (7.12)	54 (3.34)	13 (0.77)	492 (10.78)	230 (5.07)	87 (1.96)	
Normal	1,008 (63.56)	751 (46.50)	540 (31.86)	2,930 (64.23)	2,434 (53.62)	1,693 (38.04)	
Overweight	378 (23.83)	610 (37.77)	791 (46.67)	966 (21.17)	1,442 (31.77)	1875 (42.13)	
Obesity	87 (5.49)	200 (12.38)	351 (20.71)	174 (3.81)	433 (9.54)	795 (17.87)	
Place of residence							<0.001
Urban	667 (42.06)	826 (51.15)	900 (53.10)	1,410 (30.91)	1,646 (36.26)	1796 (40.36)	
Rural	919 (57.94)	789 (48.85)	795 (46.90)	3,152 (69.09)	2,893 (63.74)	2,654 (59.64)	
Education							<0.001
Primary school or below	1,015 (64.00)	977 (60.50)	967 (57.05)	3,566 (78.17)	3,436 (75.70)	3,257 (73.19)	
Middle school	355 (22.38)	388 (24.02)	440 (25.96)	702 (15.39)	754 (16.61)	798 (17.93)	
High school or above	216 (13.62)	250 (15.48)	288 (16.99)	294 (6.44)	349 (7.69)	395 (8.88)	
Marital status							< 0.001
Living with spouse	1,470 (92.69)	1,500 (92.88)	1,592 (93.92)	3,995 (87.57)	4,013 (88.41)	3,944 (88.63)	
Other status	116 (7.31)	115 (7.12)	103 (6.08)	567 (12.43)	526 (11.59)	506 (11.37)	
Income (CNY)							<0.001
<4,000	305 (19.23)	269 (16.66)	249 (14.69)	1,012 (22.18)	1,044 (23.00)	915 (20.56)	
4,000–9,999	352 (22.19)	342 (21.18)	352 (20.77)	1,155 (25.32)	1,003 (22.10)	965 (21.69)	
10,000–19,999	348 (21.94)	349 (21.61)	378 (22.30)	894 (19.60)	929 (20.47)	952 (21.39)	
≥20,000	331 (20.87)	455 (28.17)	497 (29.32)	544 (11.92)	673 (14.83)	779 (17.51)	
Missing	250 (15.76)	200 (12.38)	219 (12.92)	957 (20.98)	890 (19.61)	839 (18.85)	
Smoking							<0.001
Never	853 (53.78)	952 (58.95)	1,004 (59.23)	2,645 (57.98)	2,878 (63.41)	2,903 (65.24)	
Former	205 (12.93)	227 (14.06)	258 (15.22)	519 (11.38)	471 (10.38)	455 (10.22)	
Current	528 (33.29)	436 (27.00)	433 (25.55)	1,398 (30.64)	1,190 (26.22)	1,092 (24.54)	
Alcohol drinking							<0.001
No	995 (62.74)	1,134 (70.22)	1,211 (71.45)	3,300 (72.34)	3,594 (79.18)	3,588 (80.63)	
Yes	591 (37.26)	481 (29.78)	484 (28.55)	1,262 (27.66)	945 (20.82)	862 (19.37)	
Physical activity							<0.001
Low	625 (39.41)	631 (39.07)	674 (39.76)	1851 (40.57)	1889 (41.62)	1926 (43.28)	
Moderate	252 (15.89)	317 (19.63)	365 (21.53)	833 (18.26)	890 (19.61)	912 (20.49)	
High	709 (44.70)	667 (41.30)	656 (38.70)	1878 (41.17)	1760 (38.78)	1,612 (36.22)	
Family history of hypertension							<0.001
No	1,225 (77.24)	1,171 (72.51)	1,191 (70.27)	3,823 (83.80)	3,664 (80.72)	3,471 (78.00)	
Yes	361 (22.76)	444 (27.49)	504 (29.73)	739 (16.20)	875 (19.28)	979 (22.00)	
Family history of diabetes							<0.001
No	1,518 (95.71)	1,492 (92.38)	1,556 (91.80)	4,443 (97.39)	4,313 (95.02)	4,203 (94.45)	
Yes	68 (4.29)	123 (7.62)	139 (8.20)	119 (2.61)	226 (4.98)	247 (5.55)	
HDC							<0.001
No	1,544 (97.35)	1,529 (94.67)	1,541 (90.91)	4,417 (96.82)	4,310 (94.95)	4,011 (90.13)	
Yes	42 (2.65)	86 (5.33)	154 (9.09)	145 (3.18)	229 (5.05)	439 (9.87)	

### 3.2 Association of AIP and DII with HDC

**Figure 1:**
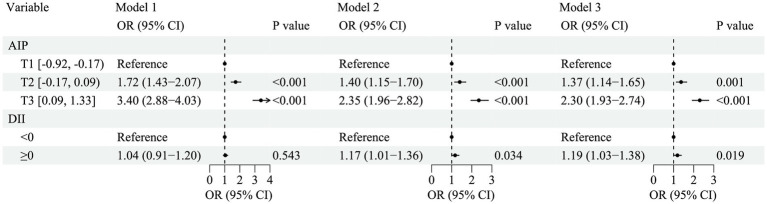
Association of atherogenic index of plasma and dietary inflammation index with hypertension–diabetes comorbidity. Model 1 was not adjusted. Model 2 was adjusted for age, sex, BMI, place of residence, education, marital status, and income. Model 3 was adjusted for smoking, alcohol drinking, physical activity, family history of hypertension, and family history of diabetes on the basis of model 2. AIP, atherogenic index of plasma; DII, dietary inflammatory index; OR, odds ratio; CI, confidence interval; T, tertiles.

**Figure 2:**
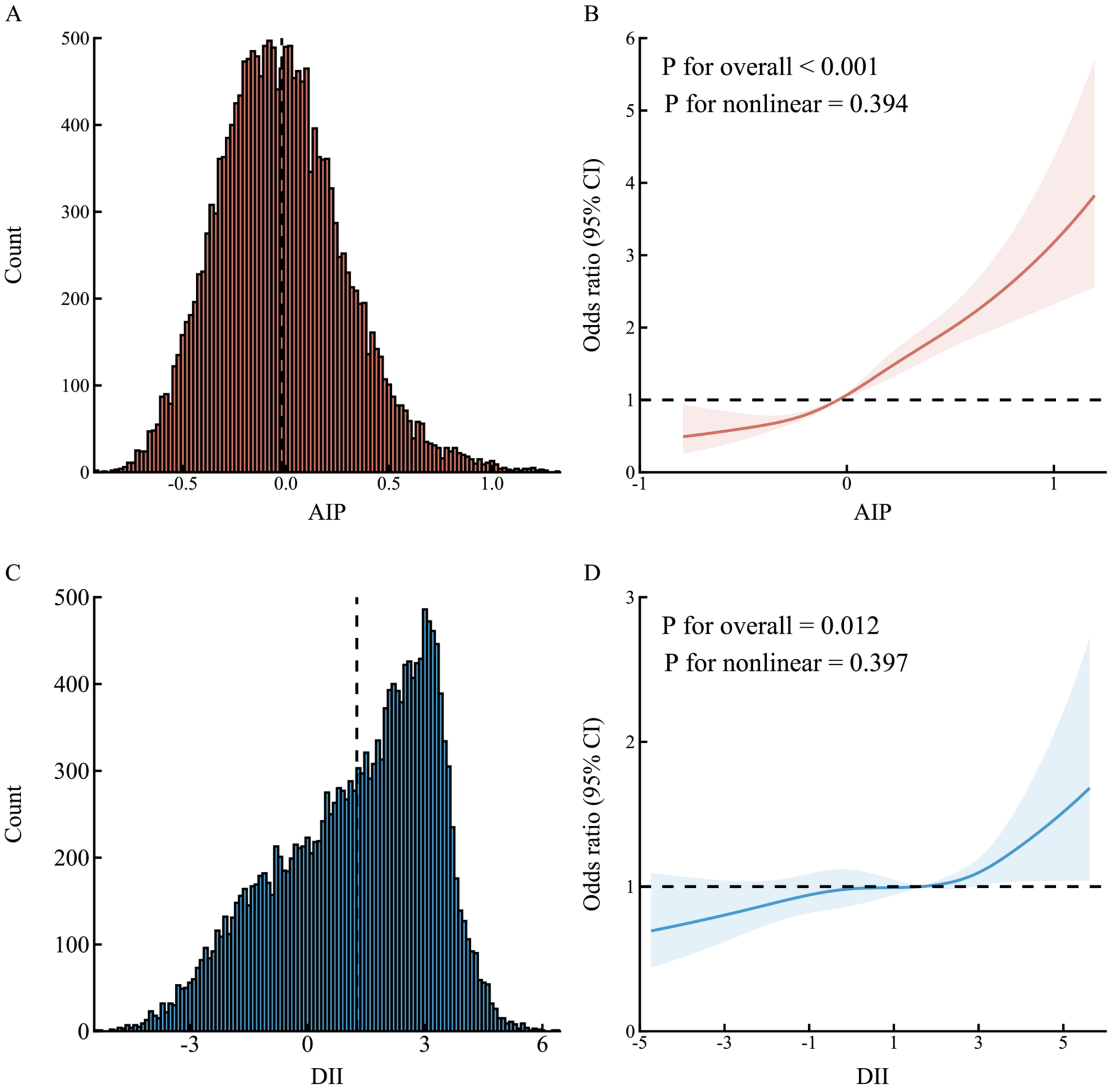
Association of atherogenic index of plasma and dietary inflammatory index with hypertension–diabetes comorbidity. **(A,C)** Distribution of AIP and DII; **(B,D)** adjusted for age, sex, BMI, place of residence, education, marital status, income, smoking, alcohol drinking, physical activity, family history of hypertension, and family history of diabetes. AIP, atherogenic index of plasma; DII, dietary inflammatory index; CI, confidence interval.

Figure 1 illustrates the relationship between AIP, DII, and HDC. After adjusting for confounders (Model 3), compared with the T1 group of AIP, the risk of HDC increased by 0.37-fold (OR = 1.37, 95% CI: 1.14–1.65, *p* = 0.001) in the T2 group and by 1.30-fold (OR = 2.30, 95% CI: 1.93–2.74, *p* < 0.001) in the T3 group; compared with the anti-inflammatory diet group (DII < 0), the risk of HDC increased by 0.19-fold (OR = 1.19, 95% CI: 1.03–1.38, *p* = 0.019) in the pro-inflammatory diet group (DII ≥ 0). RCS analysis results showed a linear positive correlation between AIP (*P* for non-linear = 0.394; Figures 2A,B), DII (*P* for non-linear = 0.397; Figures 2C,D), and HDC risk.

### 3.3 Interaction and joint effects of AIP and DII on HDC

**Figure 3:**
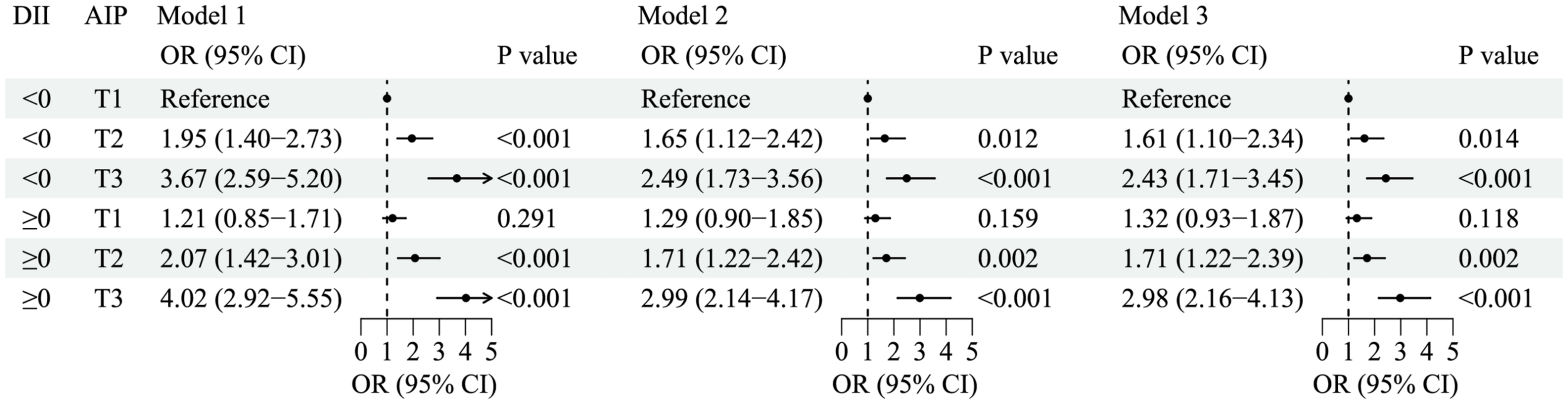
Joint associations of atherogenic index of plasma and dietary inflammatory index with hypertension–diabetes comorbidity. Model 1 was not adjusted. Model 2 was adjusted for age, sex, BMI, place of residence, education, marital status, and income. Model 3 was adjusted for smoking, alcohol drinking, physical activity, family history of hypertension, and family history of diabetes on the basis of model 2. AIP, atherogenic index of plasma; DII, dietary inflammatory index; OR, odds ratio; CI, confidence interval; T, tertiles.

No significant multiplicative (OR = 0.94, 95% CI: 0.63–1.40) and additive (RERI = 0.25, 95% CI: −0.37 − 0.87) interactions were found between AIP and DII on the risk of HDC (Table 2). Figure 3 shows the joint association of AIP and DII with HDC. After adjusting for confounders (Model 3), compared with the group with an anti-inflammatory diet and lower AIP, the group with a pro-inflammatory diet and higher AIP had a 1.98-fold (OR = 2.98, 95% CI: 2.16–4.13, *p* < 0.001) increased risk of HDC.

**Table 2:** Interactive effects of atherogenic index of plasma and dietary inflammatory index on hypertension–diabetes comorbidity.

Interactive items	Interactive effects (95% CI)		
	Model 1	Model 2	Model 3
Additive effects			
RERI	0.14 (−0.64–0.94)	0.22 (−0.41–0.84)	0.25 (−0.37–0.87)
AP	0.04 (−0.16–0.24)	0.07 (−0.14–0.28)	0.08 (−0.13–0.29)
SI	1.05 (0.79–1.39)	1.12 (0.78–1.59)	1.14 (0.79–1.63)
Multiplicative effect	0.91 (0.61–1.35)	0.93 (0.62–1.39)	0.94 (0.63–1.40)

Model 1 was not adjusted. Model 2 was adjusted for age, sex, BMI, place of residence, education, marital status, and income. Model 3 was adjusted for smoking, alcohol drinking, physical activity, family history of hypertension, and family history of diabetes on the basis of model 2.

RERI, relative excess risk due to interaction; AP, attributable proportion due to interaction; SI, synergy index; CI, confidence interval.

### 3.4 Subgroup and sensitivity analyses

Results stratified by sex were consistent with the main findings (Tables 3, 4). Sensitivity analyses using DII tertiles (T1: DII < 0.54; T2: 0.54 ≤ DII < 2.48; T3: DII ≥ 2.48) yielded results consistent with the primary analysis using DII binary classification (Tables 5–7).

**Table 3:** Interactive effects of atherogenic index of plasma and dietary inflammatory index on hypertension–diabetes comorbidity by sex.

Interactive items	Interactive effects (95% CI)	
	Male	Female
Additive effects		
RERI	0.31 (−0.58–1.19)	0.23 (−0.63–1.09)
AP	0.09 (−0.18–0.36)	0.08 (−0.25–0.41)
SI	1.15 (0.73–1.82)	1.15 (0.62–2.12)
Multiplicative effect	0.96 (0.57–1.62)	0.94 (0.50–1.77)

Adjusted for age, BMI, place of residence, education, marital status, income, smoking, alcohol drinking, physical activity, family history of hypertension, and family history of diabetes.

RERI, relative excess risk due to interaction; AP, attributable proportion due to interaction; SI, synergy index; CI, confidence interval.

**Table 4:** Joint associations of atherogenic index of plasma and dietary inflammatory index with hypertension–diabetes comorbidity by sex.

DII	AIP	Male		Female	
		OR (95% CI)	*P-*value	OR (95% CI)	*P*-value
<0	T1	Reference		Reference	
<0	T2	1.66 (1.08–2.57)	0.022	1.34 (0.72–2.49)	0.360
<0	T3	2.71 (1.73–4.23)	<0.001	2.11 (1.18–3.75)	0.012
≥0	T1	1.26 (0.81–1.98)	0.305	1.39 (0.79–2.44)	0.260
≥0	T2	1.86 (1.16–2.99)	0.011	1.71 (1.00–2.95)	0.052
≥0	T3	3.28 (2.16–4.97)	<0.001	2.68 (1.58–4.56)	<0.001

Adjusted for age, BMI, place of residence, education, marital status, income, smoking, alcohol drinking, physical activity, family history of hypertension, and family history of diabetes.

AIP, atherogenic index of plasma; DII, dietary inflammatory index; OR, odds ratio; CI, confidence interval; T, tertiles.

**Table 5:** Sensitivity analysis for the association between dietary inflammatory index and hypertension–diabetes comorbidity.

Variable	Model 1		Model 2		Model 3	
	OR (95%CI)	*p*-value	OR (95%CI)	*P*-value	OR (95%CI)	*P*-value
DII						
T1 [−5.43, 0.54)	Reference		Reference		Reference	
T2 [0.54, 2.48)	0.95 (0.82–1.10)	0.489	1.02 (0.88–1.19)	0.771	1.03 (0.89–1.21)	0.676
T3 [2.48, 6.37]	1.01 (0.87–1.17)	0.908	1.19 (1.02–1.38)	0.032	1.20 (1.02–1.41)	0.025

Model 1 was not adjusted. Model 2 was adjusted for age, sex, BMI, place of residence, education, marital status, and income. Model 3 was adjusted for smoking, alcohol drinking, physical activity, family history of hypertension, and family history of diabetes on the basis of model 2.

DII, dietary inflammatory index; OR, odds ratio; CI, confidence interval; T, tertiles.

**Table 6:** Sensitivity analysis for the interactive effects of atherogenic index of plasma and dietary inflammatory index on hypertension–diabetes comorbidity.

Interactive items	Interactive effects (95% CI)		
	Model 1	Model 2	Model 3
Additive effects			
RERI	0.21 (−0.57–0.99)	0.42 (−0.22–1.07)	0.44 (−0.20–1.09)
AP	0.06 (−0.16–0.28)	0.15 (−0.07–0.36)	0.15 (−0.07–0.37)
SI	1.09 (0.78–1.52)	1.28 (0.83–1.98)	1.31 (0.84–2.04)
Multiplicative effect	1.02 (0.68–1.53)	1.09 (0.72–1.64)	1.08 (0.72–1.64)

By constructing interaction terms DII (T1, T3) and AIP (T1, T3), the interaction effects were analyzed. Model 1 was not adjusted. Model 2 was adjusted for age, sex, BMI, place of residence, education, marital status, and income. Model 3 was adjusted for smoking, alcohol drinking, physical activity, family history of hypertension, and family history of diabetes on the basis of model 2.

RERI, relative excess risk due to interaction; AP, attributable proportion due to interaction; SI, synergy index; CI, confidence interval.

**Table 7:** Sensitivity analysis for the joint associations of atherogenic index of plasma and dietary inflammatory index with hypertension–diabetes comorbidity.

DII	AIP	Model 1		Model 2		Model 3	
		OR (95% CI)	*P-*value	OR (95% CI)	*P*-value	OR (95% CI)	*P*-value
T1	T1	Reference		Reference		Reference	
T2	T1	0.92 (0.64–1.33)	0.672	0.97 (0.67–1.41)	0.887	0.98 (0.68–1.41)	0.900
T3	T1	1.07 (0.75–1.51)	0.726	1.17 (0.82–1.67)	0.395	1.19 (0.83–1.69)	0.343
T1	T2	1.84 (1.34–2.53)	<0.001	1.47 (1.07–2.04)	0.019	1.43 (1.04–1.97)	0.029
T2	T2	1.60 (1.15–2.21)	0.005	1.37 (0.98–1.91)	0.063	1.34 (0.97–1.86)	0.078
T3	T2	1.71 (1.24–2.36)	0.001	1.54 (1.11–2.14)	0.010	1.54 (1.11–2.13)	0.009
T1	T3	3.27 (2.44–4.39)	<0.001	2.21 (1.63–2.99)	<0.001	2.15 (1.60–2.90)	<0.001
T2	T3	3.37 (2.51–4.53)	<0.001	2.41 (1.78–3.25)	<0.001	2.40 (1.78–3.23)	<0.001
T3	T3	3.55 (2.64–4.76)	<0.001	2.77 (2.05–3.75)	<0.001	2.73 (2.03–3.69)	<0.001

Model 1 was not adjusted. Model 2 was adjusted for age, sex, BMI, place of residence, education, marital status, and income. Model 3 was adjusted for smoking, alcohol drinking, physical activity, family history of hypertension, and family history of diabetes on the basis of model 2.

AIP, atherogenic index of plasma; DII, dietary inflammatory index; OR, odds ratio; CI, confidence interval; T, tertiles.

## 4 Discussion

This nationwide cross-sectional study investigated the interacting and joint effects of AIP and DII on hypertension-diabetes comorbidity risk in Chinese elderly adults. Our findings showed that individuals with both risk factors exhibited a markedly higher HDC risk, despite no significant interactions being detected. This offers important implications for comprehensive prevention strategies targeting both lipid metabolism and dietary inflammation in the elderly population.

Insulin resistance (IR) is the common pathophysiological basis for both diabetes and hypertension, and plays a crucial role in the occurrence and development of these two diseases (2). The AIP has been established in previous studies as a reliable surrogate marker of IR (32–34). AIP is calculated as the logarithm of the TG and HDL-C ratio (10). Elevated TG levels can induce lipotoxicity, thereby promoting the occurrence and development of IR (35). HDL-C can influence glucose homeostasis through mechanisms such as promoting insulin secretion, improving insulin sensitivity, and promoting non-insulin-dependent glucose uptake; reduced HDL-C levels exacerbate IR (36). Additionally, elevated AIP is closely associated with endothelial dysfunction, impairing vascular function by reducing nitric oxide bioavailability and disrupting the balance between vasodilation and vasoconstriction (11). The chronic low-grade inflammatory state accompanying elevated AIP, characterized by increased levels of tumor necrosis factor-*α* (TNF-α) and interleukin-6 (IL-6), can further damage vascular endothelial function and contribute to hypertension (37). Elevated TG levels increase free fatty acid release, stimulating pancreatic α cells to secrete glucagon (35, 38), which plays a key role in diabetes pathogenesis by regulating hepatic glucose output, energy metabolism, and neural signaling pathways (39, 40). Meanwhile, reduced HDL-C levels impair cholesterol efflux function, exacerbating systemic inflammation and promoting insulin resistance and glucose metabolism disorders (41). Zhou et al. (11), in a cross-sectional analysis based on the China Health and Retirement Longitudinal Study (CHARLS) database (*n* = 8,450, mean age 59.57 ± 9.41 years), found a positive correlation between AIP and HDC risk; for each unit increase in AIP, the risk of HDC increased by 2.75-fold. Chen et al. (12), in a cross-sectional analysis of 5,285 adults from the US National Health and Nutrition Examination Survey (NHANES) database, also found a positive correlation between AIP and HDC risk (OR = 3.95, 95% CI: 1.66–9.39). This study found a significant positive correlation between AIP and HDC risk in Chinese elderly adults, consistent with the above findings.

Chronic inflammation plays an important role in the development of hypertension and diabetes (1, 3). Previous studies have shown that elevated levels of inflammatory markers such as C-reactive protein (CRP) and interleukin-6 (IL-6) are associated with risks of hypertension and diabetes (42–44). The body’s inflammatory status is influenced by various factors, with diet being one of the modifiable lifestyle factors (13). A meta-analysis including 22 randomized controlled trials indicated that adherence to a Mediterranean diet rich in vegetables, fruits, whole grains, and olive oil significantly reduced levels of inflammatory markers such as IL-6, Interleukin-1beta (IL-1β), and CRP (45). Conversely, the Western diet, characterized by high calories, fat, sugar, and low dietary fiber, is associated with elevated levels of multiple inflammatory markers (46). DII is an effective tool for assessing the inflammatory potential of diet and is significantly correlated with various inflammatory markers (47). Patel et al. (48), based on NHANES (1999–2018), found that among individuals aged 60 and above, compared to the lowest quartile of DII, the highest quartile had a 19 and 17% increased risk of hypertension and diabetes, respectively. A study on individuals aged 55 and above in northern China also showed that the highest quartile of DII had a 28 and 23% increased risk of hypertension and hyperglycemia, respectively, compared to the lowest quartile (49). Furthermore, a meta-analysis found that higher DII was significantly associated with increased risks of hypertension, hyperglycemia, and metabolic syndrome (50). The results of this study are consistent with the above evidence, showing that the pro-inflammatory diet group (DII ≥ 0) had a 19% increased risk of HDC compared to the anti-inflammatory diet group (DII < 0) among Chinese elderly adults.

This study did not find significant multiplicative or additive interactions between AIP and DII on HDC risk. AIP, as a composite marker reflecting lipid metabolism disorders, contributes to the development of hypertension and diabetes primarily through pathways involving insulin resistance, endothelial dysfunction, and glucagon secretion (35, 51–53). DII, as an assessment tool for dietary inflammatory potential, exerts its effects mainly through mechanisms such as activating inflammatory signaling pathways and inducing oxidative stress (1, 54, 55). Although these two pathways intersect, as lipid metabolism disorders can trigger inflammatory responses and inflammatory states can exacerbate lipid metabolism abnormalities, they may act on different nodes along the disease development continuum. Heidarzadeh-Esfahani et al. (18) demonstrated that compared with the lowest quartile of DII, participants in the highest quartile had modestly elevated AIP levels (*β* = 0.06, 95% CI: 0.02–0.10), indicating a positive correlation between the two factors, albeit with a weak association strength. The synergistic effect may not have reached the threshold for statistically significant interaction. Furthermore, HDC is influenced by multiple factors, including genetics, lifestyle, and environmental factors (3, 56). The complex interrelationships among these factors may, to some extent, obscure the potential interaction effects between AIP and DII.

Additionally, joint effect analysis in this study showed that individuals with both high AIP and a pro-inflammatory diet had a significantly higher HDC risk than those exposed to only a single risk factor. Previous studies have shown that DII is negatively correlated with HDL-C and positively correlated with TG and AIP (18, 57). Adherence to the DASH diet (Dietary Approaches to Stop Hypertension) can reduce TG levels in hypertensive patients (58), suggesting that dietary interventions have a positive effect on improving AIP. Moreover, previous research suggests a certain joint effect between lipid metabolism and dietary inflammation. For example, DII mediated 7.24% of the association between non-HDL-C/HDL-C and chronic obstructive pulmonary disease (59); a cohort study showed that the triglyceride-glucose index (TyG) mediated 5.90 and 9.35% of the associations between DII and non-fatal cardiovascular disease and stroke, respectively (60); DII may indirectly increase the risk of cardiovascular disease by elevating TyG and reducing HDL-C levels (61, 62). Therefore, when formulating prevention and control strategies for HDC, emphasis should be placed on combining lipid management with dietary regulation to more effectively reduce HDC risk through comprehensive interventions.

This study, utilizing nationally representative data from China, validated the effects of DII and AIP on hypertension-diabetes comorbidity in Chinese elderly adults, and for the first time, explored the interactive and joint effects of DII and AIP on HDC. Nevertheless, the study possessed certain shortcomings. First, as a cross-sectional study, it cannot determine causality, which requires further research for validation. Second, to avoid the potential impact of dietary interventions on the results, the study excluded patients with hypertension, diabetes, or dyslipidemia undergoing dietary interventions, limiting the generalizability of the findings to such patient populations. Third, limited by the coverage of the Chinese Food Composition Table, DII calculation was based on 22 dietary components, which may not fully reflect the overall inflammatory potential of the diet. However, previous studies have shown that even if fewer than 30 nutrients are considered in the calculation, the DII remains valid (16, 23, 24, 63). Additionally, dietary data were collected through a food frequency questionnaire, and problems such as recall bias and reporting bias may exist during the survey.

## 5 Conclusion

Higher AIP levels and a pro-inflammatory diet are associated with an increased risk of hypertension-diabetes comorbidity in Chinese elderly adults. Although no significant multiplicative or additive interaction between AIP and DII on HDC risk was observed, joint effect analysis showed that individuals with both high AIP and a pro-inflammatory diet had a significantly higher HDC risk than those with only a single risk factor. Therefore, when formulating prevention and control strategies for HDC in the elderly, simultaneous attention should be paid to both lipid metabolism disorders and dietary inflammatory levels to more effectively reduce the risk of HDC through comprehensive interventions.

## Data Availability

The data analyzed in this study was obtained from China Nutrition and Health Surveillance conducted by the Chinese Center for Disease Control and Prevention (China CDC), due to administrative restrictions, the data is not publicly available. Requests to access these datasets should be directed to the China CDC via www.chinanutri.cn.
